# Microbiota–gut–brain axis in health and neurological disease: Interactions between gut microbiota and the nervous system

**DOI:** 10.1111/jcmm.70099

**Published:** 2024-09-19

**Authors:** Yuhong He, Ke Wang, Niri Su, Chongshan Yuan, Naisheng Zhang, Xiaoyu Hu, Yunhe Fu, Feng Zhao

**Affiliations:** ^1^ Department of Operating Room China‐Japan Union Hospital of Jilin University Changchun Jilin China; ^2^ Department of Clinical Veterinary Medicine College of Veterinary Medicine, Jilin University Changchun Jilin China

**Keywords:** gut microbiota, microbiota–gut–brain axis, nervous system, neurotransmitter

## Abstract

Along with mounting evidence that gut microbiota and their metabolites migrate endogenously to distal organs, the ‘gut–lung axis,’ ‘gut–brain axis,’ ‘gut–liver axis’ and ‘gut–renal axis’ have been established. Multiple animal recent studies have demonstrated gut microbiota may also be a key susceptibility factor for neurological disorders such as Alzheimer's disease, Parkinson's disease and autism. The gastrointestinal tract is innervated by the extrinsic sympathetic and vagal nerves and the intrinsic enteric nervous system, and the gut microbiota interacts with the nervous system to maintain homeostatic balance in the host gut. A total of 1507 publications on the interactions between the gut microbiota, the gut–brain axis and neurological disorders are retrieved from the Web of Science to investigate the interactions between the gut microbiota and the nervous system and the underlying mechanisms involved in normal and disease states. We provide a comprehensive overview of the effects of the gut microbiota and its metabolites on nervous system function and neurotransmitter secretion, as well as alterations in the gut microbiota in neurological disorders, to provide a basis for the possibility of targeting the gut microbiota as a therapeutic agent for neurological disorders.

## 1 INTRODUCTION

The majority of microbial interactions with the body occur in the intestinal tract, ‘gut microbiota’ refers to the many microbial communities that live there. More than 1000 different species of bacteria make up the enormous and varied gut microbiota,^1^ with the *Firmicutes*/*Bacteroidetes* being the two most prevalent phyla.^2^ Studies have shown that there is a complex network of interactions between the gut microbiota and the host, which in turn maintains homeostasis.^3^ Gut homeostasis is a dynamic equilibrium condition that is maintained by the interactions between the host, gut microbiota, diet and metabolites. A generally steady intestinal homeostasis is required for maintaining the normal structure and physiological function of the intestine, inhibiting the colonization of pathogenic microorganisms and regulating host immunological response. However, alterations in external conditions or inappropriate and excessive use of antibiotics induce the dysbiosis of gut microbiota, primarily in the form of increased pathogenic bacteria or insufficient probiotics, triggering the secretion of amyloid and lipopolysaccharides (LPS), increasing intestinal permeability and allowing other cytokines like LPS to penetrate the intestinal wall, triggering toll‐like receptors (TLR) and releasing inflammatory cytokines.^4^ Recent studies have shown that gut microbial dysbiosis not only induces gastrointestinal symptoms, but even impairs brain function, resulting in cognitive impairment, mood and behavioural changes.^5^

The gut–brain axis primarily consists of the central nervous system (CNS), enteric nervous system (ENS), autonomic nervous system (ANS) and hypothalamic–pituitary–adrenal axis (HPA).^6^ A brand‐new idea, the microbiota–gut–brain axis (MGB), has been put forth since the microbiota is crucial to the communication between the gut and the brain.^4^ Through the gut–brain axis, the microbiota affects the CNS, and vice versa. Studies have shown that inflammatory factors produced by gut dysbiosis increase the permeability of the blood–brain barrier (BBB),^7^ which in turn penetrates brain tissue and induces neuroinflammation, while probiotics, on the contrary, prevent inflammatory cytokines from entering the blood circulation and lessen the harm to the nervous system.^8^ Also, the composition and abundance of the gut microbiota significantly altered in people with neurological diseases as Parkinson's disease (PD),^9^ depression^10^ and Alzheimer's disease (AD).^11^ The gut microbiota communicate with the brain via three parallel and interacting pathways^12^: (1) neural pathway; (2) immune pathway; (3) endocrine pathway. Although there are three parallel and related pathways for the MGB, this review focuses on the interaction between the neural pathway and the gut microbiota, with a providing a rationale for targeting the gut microbiota for the prevention and treatment of neurological disorders.

## 2 THE MICROBIOTA–GUT–BRAIN AXIS

In the 1880s, William James, an American psychologist, and Carl Lange, a Danish physiologist, initially proposed the hypothesis that the brain and the gut communicate in both directions. However, the concept of the gut–brain axis was not fully developed until the late 19th and early 20th centuries. Currently, the MGB axis represents an interaction network of gut microbiota, microbiota‐related metabolites, intestinal cells, multi‐level nervous system and neurohumoral pathways.^13^ There are two basic types of methodologies utilized to research the interplay of the MGB axis: (1) studies utilizing germ‐free (GF) animals, followed by faecal microbiota transplantation (FMT) to examine the influence of normal gut microbiota or particular intestinal bacteria on MGB axis^14^, ^15^; (2) mice with gut microbiota dysbiosis or the use of probiotic for mice exhibiting neurological symptoms to evaluate whether there is an improvement in symptoms, afterwards laterally validating the effect of microbiota on MGB axis.^16^

**FIGURE 1:**
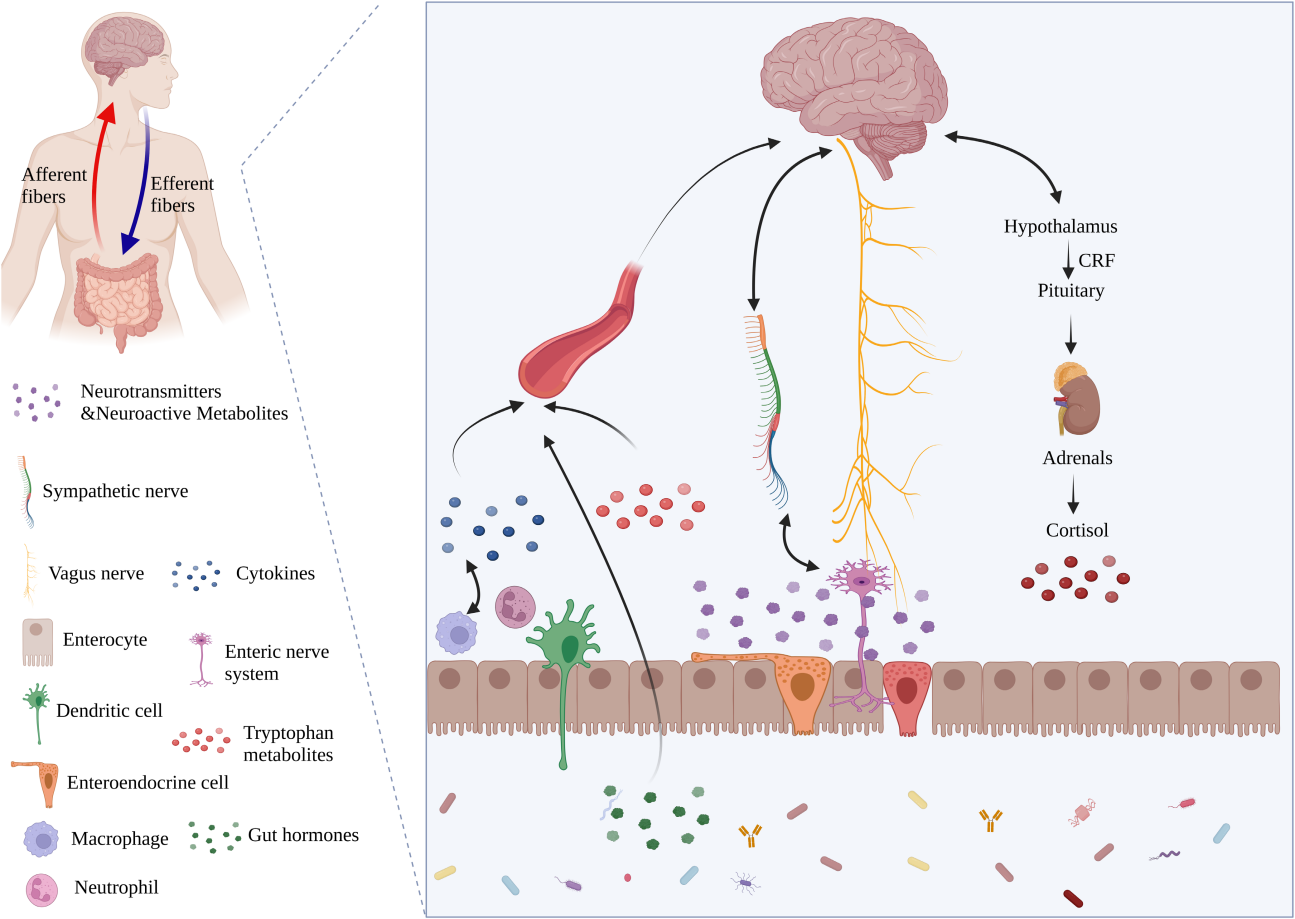
Three pathways between the microbiota–gut–brain axis. The gut microbiota and the CNS communicate via at least three parallel and related pathways. The gut microbiota potentially metabolizes and produce neurotransmitters, neuroactive substances, SCFAs and other substances that influence brain function and development by crossing the BBB. Moreover, there is a direct neuronal connection from the gut to the brain known as the VN, which is able to sense messages emitted by enteric neurons to directly affect brain function. Neurotransmitters or hormones enter the bloodstream alongside cytokines released by immune cells and penetrate the BBB, impacting the mood, cognition and behaviour. Schematic created with BioRender.com.

The relationship between the gut microbiota and the brain involves a number of complex pathways, including immunological, neurological and endocrine pathway (Figure 1).^17^ Because 70%–80% of immune cells in animals are located in lymphoid tissues linked with the colon, changes in the gut microbiota directly affect the intestinal immune system and provide a timely immunological response,^18^ which has a significant impact on the CNS.^19^ The interaction between the gut microbiota and the host controls the local immune response and releases cytokines into the bloodstream that disrupt the BBB and have a direct impact on brain function. Moreover, gut microbiota influences the number and function of microglia in the brain, which in turn influences brain function.^20^ In addition to immune pathways, gut microbiota and their metabolites, such as indole derivatives, serotonin, γ‐aminobutyric acid (γ‐GABA), secondary bile acids and short‐chain fatty acid (SCFAs), may control the production of neuropeptides via endothelial cell and enteroendocrine cells (EECs) or may directly release neurotransmitters and neuromodulators, which may affect brain function.^21^, ^22^ Moreover, the neural pathway directly connects the brain to the gut and consists mainly of the sympathetic nerve (SN), the vagus nerve (VN) and the ENS. The entire gut is innervated by a dense neural network, including the intrinsic ENS (up to 200–500 million neurons) and the extrinsic VN, SN, spinal and sensory nociceptive nerves. Multiple reflex circuits formed between the external and internal nerves innervating the intestine, synaptic connections between the extrinsic nerves and the dorsal horn neurons of the spinal cord and the nucleus of solitary tract of VN can transmit a variety of information from the intestine to the brain.^23^

### 2.1 Sympathetic innervation of the gastrointestinal tract

Intestinal sympathetic innervation originates from prevertebral ganglia within the abdomen, including the paired celiac (CG) and superior/inferior mesenteric regions (SMG/IMG).^24^ Initial reports suggested that sympathetic fibres were infrequent in the intestinal mucosa, but as histological techniques advanced, this belief was disproved.^25^ Immunohistochemistry for norepinephrine synthase, tyrosine hydroxylase (TH) or dopamine β‐hydroxylase is now available to localize sympathetic neurons.^24^, ^26^ It is now widely believed that sympathetic nerve fibres enter the intestinal wall along the arteries and terminate in the submuscular and submucosal plexuses. In addition to entering the enteric plexus, sympathetic nervous fibres also innervate the mucosa and lymphoid tissue surrounding the colon.^27^ Generally, the sympathetic nervous system inhibits intestinal peristalsis and secretion as well as mediating inflammatory responses through catecholamines and neuropeptides.^28^ Additionally, norepinephrine (NE), a neurotransmitter released by sympathetic postganglionic fibre endings, binds to adrenergic receptors and inhibits gastric secretion and muscular activity.^29^ Furthermore, epinephrine (E) binding to adrenergic receptors surrounding macrophages proximal axons may affect cytokine levels and inflammatory responses.^30^

### 2.2 Vagal innervation of the gastrointestinal tract

The parasympathetic nerves that innervate the gastrointestinal tract are primarily derived from the VN, while those that innervate the distal colon are derived from the sacral section of the spinal cord's pelvic nerve. The VN, which serves as the tenth brain nerve as well as a component of the peripheral nervous system (PNS), extends from the brainstem and innervate the viscera, allowing them to be the fastest and most direct pathway of communication between the gut microbiota and the brain.^4^ The VN, which has an 80% afferent fibre composition and 20% efferent fibre, has the ability to transfer information from the bottom‐up and provide feedback from the top‐down.^4^ Their preganglionic fibres replace intramural plexus neurons after entering the gastrointestinal wall, and the postganglionic fibres that follow the replacement are dispersed to the gastrointestinal wall's smooth muscle and glandular cells.^31^ The majority of postganglionic fibres are excitatory cholinergic fibres, which means that the fibre terminals release acetylcholine (Ach) and activate the effector organs. A few of them are inhibitory fibres whose ends do not produce Ach but may be peptides, so these intramural nerves are known as peptidergic nerves.^32^

### 2.3 The ENS

In 1921, Langley was the first to refer to the nerve plexus that makes up the gut wall as the ENS. The ENS is made up of enteric neurons and enteric glial cells (EGCs) and can be divided into intermuscular and submucosal plexuses, which is independent of the CNS and does not require its innervation, yet it is regulated by the SN and VN.^33^ The structure and function of enteric neurons can be roughly categorized into three groups: intrinsic primary afferent neurons that perceive inputs, intermediate neurons that relay information and motor neurons that control motor and humoral secretion, among other functions. The ENS regulates gastrointestinal motility and secretion, the biological functions of intestinal epithelial, and immune response and inflammatory processes.^34^

**FIGURE 2:**
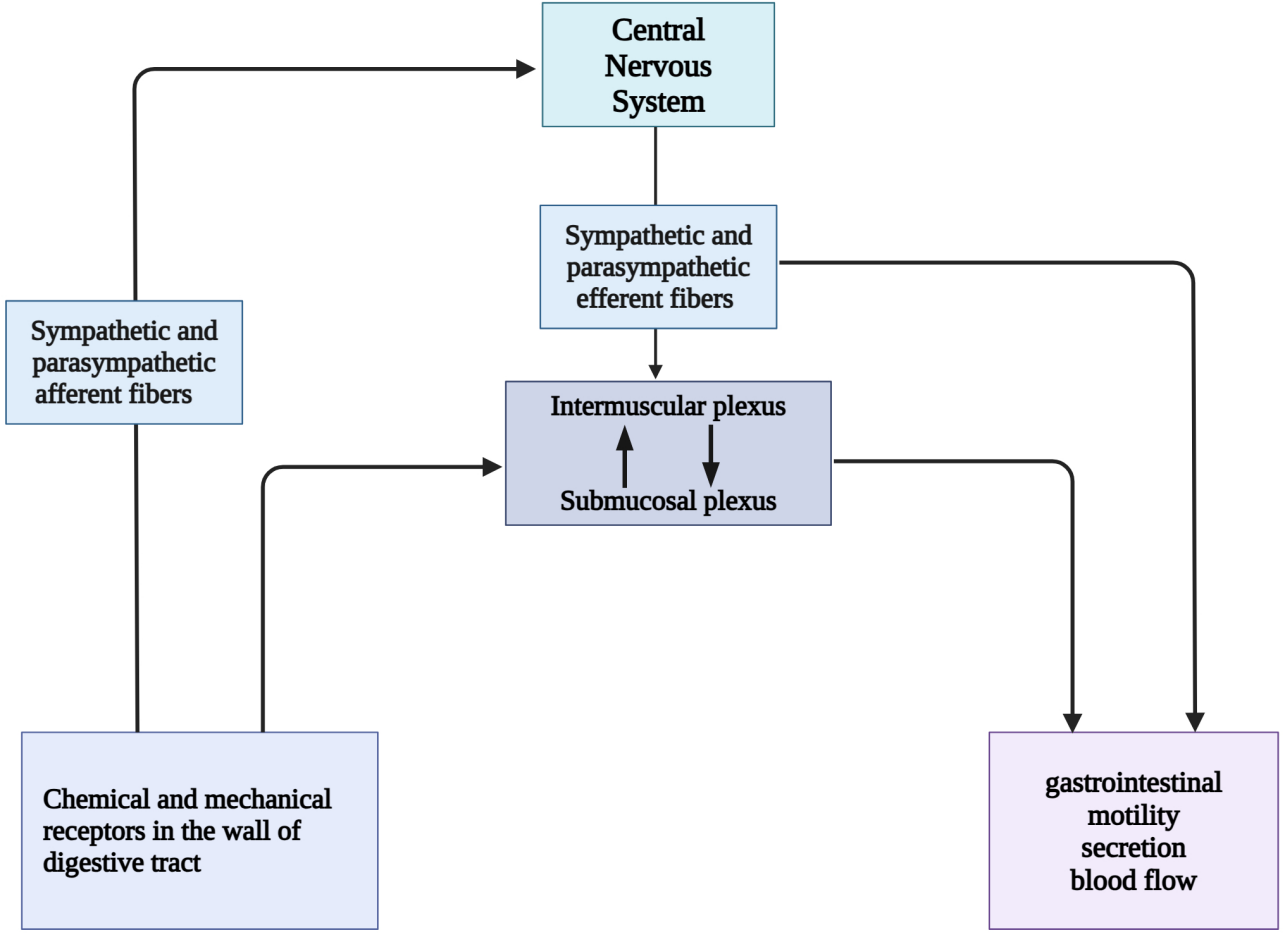
Innervation of the gastrointestinal nervous system. The intrinsic enteric nervous system and extrinsic vagus, sympathetic and spinal nerves, as well as sensory nociceptive nerves, innervate the entire gastrointestinal tract. The submucosal and intermuscular plexuses, which independently regulate gastrointestinal motility, secretion and blood flow. The activity of the gastrointestinal tract is influenced by the sympathetic and parasympathetic afferent fibres signalling to the CNS. Moreover, sympathetic and parasympathetic efferent fibres transfer information about the gastrointestinal tract to the CNS, which in turn impacts brain function. Schematic created with BioRender.com.

In conclusion, interactions between the SN, VN and ENS are necessary for the neuronal pathways of the MGB (Figure 2). Some microbial elements, microbial regulatory hormones and microbial‐dependent immune mediators interact with the ENS and its innervated vagus and spinal afferent nerves directly to transfer information to the brain and affect brain function.^35^ In turn, spinal and vagal efferent nerves send instructions from the brain to the gut to regulate gastrointestinal function.^36^ So how do the nervous system and gut microbiota interact to maintain homeostasis, and how do gut microbiota and the nervous system change in disease states? This paper aims to summarize the interactions between neurotransmitters, the nervous system and the gut microbiota in both healthy and diseased states.

### 2.4 Intestinal cells in the gut microbiota‐brain

A variety of cell types are distributed throughout the gut, including immune cells, neuronal cells and epithelial cells that constitute the gut's mucosal lining. The intestinal epithelium is a distinctive mucosal interface constituted of intestinal epithelial cells, whose monitoring and defensive functions are vital for the maintenance of gut homeostasis. Various EECs within the intestinal epithelium not only mediate physiological effects through the release of a variety of hormones, but it has also been demonstrated that there is an anatomical link between EECs and neurons.^37^ It is noteworthy that enterochromaffin cells (ECs), which are derived from basal stem cells of the intestinal epithelium, interact with nutrients, the ENS, and gut microbiota, and secrete approximately 95% of the body's 5‐hydroxytryptamine (5‐HT).^38^, ^39^ Recent studies have demonstrated that ECs cells express sensory receptors, indicating that ECs potentially function as chemoreceptors that directly perceive a diverse range of stimuli both in vivo and ex vivo.^40^ Additionally, they have the capacity to establish synaptic connections with afferent neurons to transfer information. Furthermore, it has been demonstrated that the gut microbiota significantly modulates 5‐HT synthesis in the colon. It may be postulated that this phenomenon is related to the upregulation of the expression of the rate‐limiting enzyme for 5‐HT synthesis, TPH1, by SCFAs and certain secondary bile acids.^22^, ^39^

In addition, another aspect of the role of intestinal cells in the MGB axis is mainly characterized by interactions with the ENS. Enteric neurons located in the circumflex plexus transport nerve fibres to the submucosal plexus but do not come into direct contact with immune cells.^41^ These neurons express a wide range of neuropeptides, including Neuromedin U (NMU), NE, Calcitoningenerelatedpeptide (CGRP) and so forth.^42^ It has been demonstrated that ILC2 cells localized around NMU+ neurons express NMU1 receptors at high levels.^42^ Activation of NMUR promotes the secretion of IL‐5 and IL‐13, whereas NE activates the β2AR on the surface of ILC2 to suppress type II immunity.^42^ Similarly, enteric neurons located in the submucosal plexus transport nerve fibres to the lamina propria to release vasoactive intestinal peptide (VIP), which in turn activates VIPAC2 on the surface of CCR6 + ILC3 cells, leading to the release of IL‐22 and exerting barrier protection.^43^ In addition, VIP activates VIPAC1 on the surface of CCR6 + ILC3 to promote ILC3 recruitment to the gut. In addition, the development of M cells, which is crucial during antigen sampling, is inhibited by exogenous neuronal dorsal root ganglion in the gut via CGRP.^44^ Whereas intestinal nNOS+ neurons secrete IL‐18, which in turn induces the release of antimicrobial peptides from goblet cell,^45^ Ach activates mAchR3/4 receptors on the surface of goblet cell to regulate the secretion of antimicrobial peptides and mucus.^46^

## 3 GUT MICROBIOTAS INTERACTING WITH THE NERVOUS SYSTEM UNDER PHYSIOLOGICAL CONDITIONS

GF mice are a valuable resource for studying microbiology, immunology, oncology and nutritional metabolism because they are not exposed to microorganisms from birth and have distinct developmental and physiological functions compared to specific pathogen free (SPF) mice.^47^ GF mice have also been instrumental in investigating the role of microbiota in neurodevelopment. The absence of microbiota in GF mice induces many neurobiological changes associated with several neurological disorders, including brain development, myelination, neurogenesis and microglial activation.^47^, ^48^, ^49^

### 3.1 Influence of the gut microbiota on the CNS

The development and behavioural functions of the brain is significantly influenced by gut microbiota. The most convincing evidence comes from the fact that neurodevelopmental processes are correlated with changes in maternal and neonatal gut microbiota,^50^ and that alterations in a mother's microbiota may have an impact on her offspring's microbiome, neurodevelopment and behaviour.^51^ Numerous studies have demonstrated that GF mice experience heightened prefrontal cortical myelin formation and myelin gene expression.^48^ Additionally, the hippocampus and amygdala show an increase in volume, and there is an increase in hippocampal neurogenesis.^52^, ^53^ Furthermore, GF mice have been shown to accumulate less α‐synuclein (α‐Syn), the most significant protein in PD pathogenesis.^54^ Furthermore, it is noteworthy that GF mice induce an immature phenotype in microglia, an essential regulator of neuroinflammation, leading to changes in their development and maturity.^52^, ^55^, ^56^ GF mice also have an increased permeability of the BBB, resulting in the translocation of immune cells or bacterial components into the brain and an impact on neuroinflammation.^57^ Furthermore, a number of trophic factors or proteins that affect brain development and plasticity, including brain‐derived neurotrophic factor (BDNF), synaptophysin, postsynaptic dense area protein and others, have been shown to be regulated by gut microbiota.^58^, ^59^ BDNF is a plasticity protein that stimulates neurogenesis, synapse formation and regulates synapses.^60^ It is closely related to the development of learning, memory, cognition and other bodily functions. In particular, BDNF expression was considerably downregulated in the cerebral cortex, hippocampus and hypothalamus of GF mice, as well as the mRNA expression of genes involved in synaptic plasticity.^60^

Gut microbiota not only influences how the brain develops, but it can also have an impact on how the CNS functions. Most notably, GF mice are susceptible to anxiety, but FMT eases anxiety. For instance, *Citrobacter* infection increases anxiety behaviour and the expression of the c‐Fos protein in the vagal ganglia,^61^ whereas *Lactobacillus rhamnosus* improves anxiety and depressive behaviour in stressed mice and restores γ‐GABA expression levels to normal in the hippocampus, hypothalamus and prefrontal lobes.^62^

### 3.2 Influence of the gut microbiota on the SN and VN

In addition to its direct effects on the brain, gut microbiota also modulates the SN and VN. It has been reported that gut microbiota colonization of SPF mice reverses elevated levels of intestinal epithelial ganglion (CG‐SMG) activity in GF and antibiotic‐depleted gut microbial mice, implying that gut microbiota depletion might lead to a large rise in intestinal‐exogenous sympathetic nerve activity.^63^ Moreover, transcriptome and 16S rRNA sequencing studies indicate that particular bacteria that produce SCFAs and their metabolites influence the level of intestinal sympathetic neuronal activity via modulating the expression of the neuronal transcription factor c‐Fos.^63^ Furthermore, gut microbiota also influences vagally mediated effects. It has been demonstrated that particular bacterial strains communicate with the brain and modify behaviour by vagal signals. For instance, intestinal colonization with *Campylobacter jejuni* caused anxiety‐like behaviour in mice, which was linked to higher c‐Fos expression in the vagal afferent brain areas.^64^ It has also been demonstrated that, if the VN structure is intact, *Bifidobacterium longum/Lactobacillus rhamnosus* reduces anxiety states in mice with colitis.^65^, ^66^ Likewise, *Lactobacillus reuteri* improved social impairments in mice with autism spectrum disorders by raising the level of oxytocin in the paraventricular nucleus via stimulating the VN.^67^ In addition, gut microbiota modulates the release of hormones from EECs, such as cholecystokinin (CCK) and glucagon‐like peptides (GLP), which bind to specific receptors on the afferent fibres of the VN, which in turn feed back to the brain.^68^

### 3.3 Influence of the gut microbiota on the ENS

The formation and maturity of the ENS depend heavily on the gut microbiota. With the establishment of the gut microbiota, the ENS was developed during fetal life develops and matures.^69^ Many investigations have established that the network density of the jejunal and ileal interosseous plexus was dramatically reduced in GF mice compared to SPF animals, as well as the number of neurons in the interosseous ganglion, resulting in structural abnormalities of the jejunal and ileal interosseous plexus, whereas the number of neurons was restored after colonization of gut microbiota.^69^, ^70^ Recent evidence suggests that the microbiota is critical for the development and homeostasis of EGCs in the ENS, as evidenced by the fact that GF and antibiotic‐treated animals exhibit reduced numbers of EGCs.^71^ In addition, specific bacterial strains have an effect on the ENS. For example, *Lactobacillus reuteri* increased the excitability of mouse intestinal neurons^72^ and modulated the amplitude of the in vitro colonic nerve dependent rhythm complex.^73^, ^74^

### 3.4 Metabolites of the gut microbiota interact with the nervous system

Host‐microbiota interactions in the gut lead to the release of byproducts (cytokines, chemokines, and neurotransmitters) that enter into the blood, lymphatic system and brain. Altered levels of gut microbiota metabolites in diseases such as autism, mood disorders and AD are another piece of evidence that gut metabolites affect the nervous system. Patients with autism had higher levels of acetate and propionate and decreased levels of butyrate.^75^ Similarly, a significant reduction in the composition of butyrate‐producing gut microbiota was found in depressed patients.^76^ Transplantation of gut microbiota from senescent mice (24 months old) to the GF young mice (5–6 weeks old) for 8 weeks promoted neuronal regeneration in mice, and further studies revealed that this effect was mainly mediated by butyrate‐producing microorganisms, suggesting that butyrate has a stimulatory effect on neural regeneration.^77^ Numerous studies have shown that tryptophan metabolism is a key metabolic pathway in the MGB. There are three main metabolic pathways of tryptophan in the gastrointestinal tract: the indole and its derivatives metabolic pathway by gut microbiota, the host kynurenine oxidative degradation pathway and the host 5‐hydroxy tryptamine (5‐HT) synthesis pathway. Most tryptophan in mammals is degraded via the kynurenine pathway, and only 1% of tryptophan makes it to the 5‐HT pathway. Tryptophan metabolic pathways and related metabolites are associated with behavioural and cognitive symptoms of neurological disorders. For example, mice receiving gut microbiota from depressed patients exhibited increased anxiety‐like behaviour and high levels of kynurenine.^78^, ^79^, ^80^ GF mice exhibits stronger anxiety‐like behaviour and serotonin levels compared to normal mice.^81^ Trpa1 is an excitatory calcium‐permeable non‐selective cation channel with important roles in nociception and neuroinflammation. Tryptophan catabolite metabolites produced by gut microbiota activate Trpa1 channels on the EECs, leading to rapid activation of VN.^21^

Both the direct developmental contribution of gut microbiota to the nervous system and the indirect protection afforded by gut microbiota are indicative of the close relationship between gut microbiota and the nervous system. In addition to the effects of gut microbiota on the nervous system described above, certain gut microbiota affects neurotransmitter production or produce neurotransmitters directly.

### 3.5 Regulation of neurotransmitters by gut microbiota and metabolites

The regulation of neurotransmitters by gut microbiota is mediated by three main pathways: (1) certain gut microbiota encode genes for enzymes required for neurotransmitter synthesis, which in turn catalyse the conversion of substrates into the corresponding neurotransmitters or precursors; (2) gut microbiota produce neuroactive compounds, for instance, *Lactobacillus* produce Ach, γ‐GABA, *Bifidobacterium* produce γ‐GABA, *Bacillus/Escherichia coli* produce NE, serotonin, dopamine and *Streptococcus/Enterococcus/Candida* produce serotonin. These neurotransmitters cross the intestinal mucosa and enter the circulation, which in turn affect the function of the CNS or directly activates the VN to transmit messages to the brain; (3) gut microbiota regulate the synthesis and release of neurotransmitters by EECs through their metabolites. In addition, neurotransmitters also affect the brain function and cognitive ability through three pathways: (1) direct action on the VN; (2) action on the ENS; (3) entry into the blood circulation and crossing the BBB. We summarize the genera of gut bacteria producing relevant neurotransmitters that are currently reviewable in the literature (Table 1). In the following, we focus on Ach, 5‐HT, γ‐GABA and NE as examples to explore the regulatory role of gut microbiota and their metabolites in the synthesis and secretion of neurotransmitters.

**TABLE 1:** Summary of neurotransmitters produced by gut bacteria.

Neurotransmitter	Crosses the BBB	Bacterial strain	References
Ach	No	*Lactobacillus plantarum*; *Bacillus acetylcholine*; *Bacillus subtilis*; *Escherichia coli*; *Staphylococcus aureus*	82, 83, 84
γ‐GABA	No	*Bifidobacterium*; *Bacteroides fragilis*; *Parabacteroides*; *Eubacterium*	85, 86, 87, 88, 89
Noradrenaline	No	*Bacillus mycoides*; *Bacillus subtilis*; *Escherichia coli* (*K‐12*); *Proteus vulgaris*; *Serratia marcescens*	90, 91
Serotonin	No	*Escherichia coli* (*K‐12*); *Hafnia alvei* (*NCIMB, 11999*); *Klebsiella pneumoniae* (*NCIMB, 673*); *Lactobacillus plantarum* (*FI8595*); *Lactococcus lactis subsp. cremoris* (*MG 1363*); *Morganella morganii* (*NCIMB, 10466*); *Streptococcus thermophilus* (*NCFB2392*)	91, 92, 93
Dopamine	No	*Bacillus cereus*; *Bacillus mycoides*; *Bacillus subtilis*; *Escherichia coli*; *Escherichia coli* (*K‐12*); *Hafnia alvei* (*NCIMB*, *11999*); *Klebsiella pneumoniae* (*NCIMB*, *673*); *Morganella morganii* (*NCIMB, 10466*); *Proteus vulgaris; Serratia marcescens*; *Staphylococcus aureus*	90, 91, 92
Histamine	No	*Citrobacter freundii*; *Enterobacter* spp.; *Hafnia alvei* (*NCIMB, 11999*); *Klebsiella pneumoniae* (*NCIMB, 673*); *Lactobacillus plantarum* (*FI8595*); *Lactobacillus hilgardii*; *Lactobacillus mali*; *Lactococcus lactis subsp. cremoris* (*MG 1363*); *Lactococcus lactissubsp. lactis* (*IL1403*); *Morganella morganii* (*NCIMB*, *10466*); *Oenococcus oeni*; *Pediococcus parvulus*; *Streptococcus thermophiles* (*NCFB2392*)	94, 95, 96, 97

#### 3.5.1 Ach

Ach is the most common cholinergic neurotransmitter in the intestinal tract and transmits excitatory signals between neurons.^98^ The biosynthesis of Ach is mainly accomplished by the interaction of choline from the systemic circulation with acetyl coenzyme A with the participation of specific choline acetyltransferases. It is then released by sympathetic preganglionic fibres, parasympathetic preganglionic and postganglionic fibres. It has been demonstrated that gut microbiota plays an important role in the synthesis and secretion of Ach. For example, there are abnormalities in neuromuscular junction function and reduced expression of serum choline (acetylcholine precursor) in GF mice.^99^ In addition, Ach in the intestine can be produced by a variety of bacteria,^100^ such as *Bacillus subtilis*, *Escherichia coli* and *Staphylococcus aureus*, especially *Staphylococcus aureus*, which induces the release of Ach from neurons and acts on intestinal epithelial cells.^101^ Studies have shown that combination therapy with *Chlorella acidophilus KABP 021*, *Lactobacillus plantarum KABP 022* and *Lactobacillus plantarum KABP 023* alleviate IBS‐associated inflammation and gut microbiota dysbiosis by producing acetate and Ach.^102^

It is noteworthy that Ach is a chemical in the brain that is closely related to learning and memory functions. In the CNS, Ach plays an important role in plasticity, arousal and feedback. Damage to the cholinergic system in the brain has been shown to be associated with memory deficits in AD.^103^

#### 3.5.2 5‐HT

5‐HT, also known as serotonin, is an inhibitory neurotransmitter involved in the regulation of the mood, energy, memory, etc. Abnormal expression of 5‐HT in the brain is associated with psychiatric disorders such as depression and anxiety.^104^ Tryptophan is the only precursor substance of 5‐HT. 90% of 5‐HT in the intestine is produced by ECs, but some bacteria also produce small amounts. Studies have shown that compared to SPF mice, GF mice have significantly lower serum 5‐HT concentrations and altered ECs morphology, while these changes are restored after colonization with gut microbiota.^105^ In addition, numerous studies in recent years have shown that gut microbiota and its metabolites affect the rate‐limiting enzymes to regulate 5‐HT levels. For example, antibiotic‐treated mice showed no change in the number of ECs but decreased Tph1 gene expression,^106^ and colonization of GF mice with *spore‐forming bacteria* upregulated colonic Tph1 expression and downregulated serotonin transporter SlC6A4 expression, increasing 5‐HT levels in the colon and blood, suggesting that factors regulating Tph1 exist in the gut and are associated with microorganisms.^107^

It is noteworthy that under normal conditions, 5‐HT is unable to cross the BBB.^108^ Nevertheless, minute quantities of free plasma tryptophan can traverse the BBB to generate 5‐HT within the brain, which in turn facilitates excitatory neurotransmission via the G protein ion channel serotonin receptors. Furthermore, elevated plasma 5‐HT levels can enhance the permeability of the BBB, temporarily allowing plasma 5‐HT to enter the central nervous system.^109^

#### 3.5.3 γ‐GABA

γ‐GABA is an inhibitory neurotransmitter, a non‐protein amino acid produced by glutamic acid decarboxylase (GAD) catalysing the decarboxylation of L‐glutamate. GAD is encoded by the gadB and gadC genes, which are contained in most gut microbiota, but the mechanisms regulating the activity of GAD in *E. coli* sources have been more comprehensively studied. Previous studies have found that *Lactobacillus*/*Bifidobacterium*/*Bacteroidetes* synthesize γ‐GABA using glutamate or the gut microbiota synthesize γ‐GABA by direct conversion of the excitatory transmitter Glu.^85^ This study also showed that the abundance of *Bacteroidetes* spp. associated with the γ‐GABA production pathway was negatively correlated with depressive features of the brain, and that γ‐GABA dysregulation is often present in depressed patients.^85^ Notably, γ‐GABA cannot cross the BBB, meaning that enteric‐derived γ‐GABA may act on the CNS by affecting the VN and ENS. It has also been demonstrated that enteric‐derived γ‐GABA is transported by GAT2 to EECs, where it binds to GABA‐A and inhibits the secretion of GLP‐1, which in turn activates the solitary tract nucleus of the brainstem and the dorsal nucleus of the vagus nerve via the VN.^110^

#### 3.5.4 NE

NE is a catecholamine bioactive substance, which is synthesized gradually from tyrosine by the action of various enzymes and is synthesized and secreted by sympathetic postganglionic neurons and adrenergic neurons in the brain. Notably, although NE can cross the placenta, it cannot cross the BBB. In the brain, NE is mainly produced by nucleus coeruleus, where the neurotransmitter precursor tyrosine is converted to dopamine and finally to NE. Among other things, dopamine is also a separate neurotransmitter, and dysregulation of the dopamine system is a feature of certain neurological disorders such as schizophrenia and PD.^111^, ^112^ The intestine synthesizes more than 50% of dopamine, and aromatic amino acid decarboxylase expressed in *staphylococcus* metabolizes L‐3,4‐dihydroxyphenylalanine substances to produce dopamine.^92^ Studies have shown that increased thermogenesis in Brandt's voles during cold acclimation is accompanied by increases in monoamine neurotransmitters such as NE, gastric hunger hormones and SCFAs and changes in cecum microbiota, while the results of FMT suggest that low temperature flora elevates levels of SCFAs and its receptors and NE.^113^ In addition, *Escherichia* spp., *Bacillus* spp. and *yeast* were able to produce NE.^114^

### 3.6 Changes of gut microbiota in neurological diseases

Numerous studies have discovered major changes in the gut microbiota of people with neurological diseases (Table 2), suggesting a crucial role for the gut microbiota in neuropsychiatric disorders. For instance, clinical studies have repeatedly demonstrated that GF mice with the FMT from individuals with various psychiatric disorders exhibit the behavioural and physiological characteristic of the disorder.^115^, ^116^, ^117^ Moreover, research have indicated that the majority of individuals with neurological diseases had large reductions in gut microbiota richness, and changes in composition, but no significant variations in Shannon and Simpson indices.^118^, ^119^

**TABLE 2:** Changes in gut microbiota in patients with neurological disorders.

Diseases	Main features	Gut microbiota elevated abundance	Gut microbiota reduced abundance	References
Alzheimer's disease	Progressive cognitive impairment; memory impairment; aphasia; visuospatial impairment; executive dysfunction	*Candida*, *Proteus*; *Staphylococcus*; *Clostridium*; *Akermannia*; *Streptococcus*; *Escherichia‐Shigella*	*Bifidobacterium*; *Microbacillus*; *Bacteroides*; *Lactobacillus*; *Bifidobacterium*; *Pasteurella*; *Prevotella*; *Succinivibrio*	120, 121, 122
Autism	Social impairment, narrow interests, repetitive stereotyped behaviour Gastrointestinal symptoms: constipation, diarrhoea, flatulence	*Lactobacillus*; *Actinomyces*; *Clostridium*; *Desulfovibrio*; *Enterobacteriaceae*	*Prevotella*; *Bifidobacterium*	^123^
Parkinson's disease	Motor symptoms: resting tremor, slow movements, muscle stiffness and abnormal posture and gait Non‐motor symptoms: autonomic dysfunction, cognitive decompensation, depression, anxiety and sleep disorders Gastrointestinal symptoms: constipation, abdominal pain, diarrhoea and reflux	*Shigella* spp.; *Enterococcus* spp.; *Bacillus variabilis*; *Streptococcus* spp.	*Prevotella*; *Faecococcus* spp. *Cyanobacteria*; Genera regulating the production of SCFAs	123, 124, 125
Multiple Sclerosis	Ataxia, motor disorders, cognitive disorders	*Clostridium bolteae*; *Methanobrevibacter*; *Balch and Wolfe*; *Akkermansia*; *muciniphila*	*Methanobrevibacter* spp.; *Akkermansia* spp.; *Butyricimonas* spp.	126, 127
Major Depressive Disorder	Persistent and recurrent depressed mood, loss of interest, decreased volition and somatic symptoms	*Prevotella* spp.; *Klebsiella* spp.; *Streptococcus spp*	*Bifidobacterium*; *Lactobacillus*	^128^

## 4 INTERACTIONS BETWEEN GUT MICROBES AND THE NERVOUS SYSTEM IN NEUROLOGICAL DISEASES

Recent clinical trials and studies have shown that gut microbiota modulate the development of neurological diseases.

### 4.1 Alzheimer's disease

AD is a set of brain dysfunction syndromes produced by persistent or progressive organic damage to brain structures, with the development of amyloid plaques and inflammatory response in microglia being two of its most notable hallmarks.^11^ It is now generally accepted that patients have β‐amyloid (Aβ) deposits in the brain, which in turn induce the formation of senile plaques, Tau protein hyperphosphorylation resulting in neurofibrillary tangles (NFTs) and neuronal loss, accompanied by glial cell proliferation and eventually progressive cognitive loss and behavioural disturbances. Recent research has revealed that the abundance and composition of gut microbiota are altered in AD patients,^129^ but the order in which these changes occur in relation to pathological brain changes and the part intestinal strains play in this are still mysteries.

The current theories regarding the possible processes include: (1) gut microbial dysbiosis increase the permeability of the BBB and intestinal barrier, resulting in systemic and CNS inflammation; (2) gut microbial dysbiosis impact the secretion of neuroactive chemicals including γ‐GABA, 5‐HT, and β‐N‐methylamino‐L‐alanine; for example, research have demonstrated that *cyanobacteria* produce the neurotoxic compound β‐N‐methylamino‐L‐alanine, that causes neurological degeneration in the brain and consequently causes neurodegenerative disorders including AD and PD.^91^ (3) gut microbial dysbiosis cause an overgrowth of pathogenic bacteria.

### 4.2 Parkinson's disease

PD is the most common type of movement disorder characterized by loss of dopaminergic neurons in the substantia nigra striata, accompanied by autonomic dysfunction and psychiatric abnormalities and gut dysfunction.^9^ Constipation is the most common symptom of bowel dysfunction in PD and is usually present 10–20 years before the onset of motor symptoms, suggesting that PD may begin in the gastrointestinal tract.^130^ Misfolding of α‐Syn is a direct factor leading to dopaminergic neurons in the substantia nigra of PD patients.^131^ However, it has been shown that after injection of α‐Syn into the intestinal lining of rats, its pathway accumulates first in the enteric nerve and then retrogrades to the vagus system to reach the dorsal root motor ganglion and finally to the brain center to induce PD. similarly, vagotomy in this experiment blocked the development of PD.^132^ In addition, SPF mice overexpressing α‐syn (ASO) exhibited increased α‐syn aggregation and impaired locomotion, whereas ASO‐germ‐free mice (ASO‐GF) showed no significant α‐syn aggregation and significantly less impaired locomotion than ASO‐SPF mice.^54^ ASO‐GF mice transplanted with faeces from PD patients exhibited signs of increased dyskinesia, suggesting that microorganisms are indeed involved in α‐syn‐mediated dyskinesia.^133^

It is inferred that gut microbial dysbiosis may lead to pathological aggregation of α‐syn in the ENS by damaging the intestinal epithelial barrier, increasing intestinal permeability, exposure of pro‐inflammatory bacteria and products and inflammatory mediators to the ENS, and activation of EGCs, which in turn may spread to the CNS via the VN. In addition, gut microbiota dysbiosis leads to the BBB disruption, directly causing CNS inflammation and pathological aggregation of α‐Syn, leading to apoptosis of dopaminergic neurons.^134^ Interestingly, it has also been shown that gut microbiota dysbiosis decreases the concentration of SCFAs or affects the levels of neurotransmitters and hormones such as 5‐HT, inducing a decrease in endogenous neuroprotective factors leading to dopaminergic neuron apoptosis.

### 4.3 Autism

Approximately 40% of individuals with autism have gastrointestinal symptoms such as diarrhoea, abdominal pain and constipation, and the correlation between gastrointestinal symptoms and autism severity demonstrates the importance of the link between the gut microbiota and the brain.^135^ Investigation of stool samples from children with autism showed increased levels of *Lactobacillus*, *Clostridium* and *Desulfovibrio*.^136^ When vancomycin was used weekly to treat children with autism, it significantly improved neurological and gastrointestinal symptoms.^137^

Although numerous studies have indicated a potential association between an impaired immune system and gut microbiota in autism, the precise mechanism by which altered gut microbiota contribute to immune abnormalities in autism remains unclear. It is noteworthy that there is also evidence indicating that there is a negligible direct association between autism and gut microbiota, but a strong association with diet. Autism‐related traits promote dietary preferences, which in turn leads to a reduction in the diversity of gut microbiota and the induction of gastrointestinal problems in children with autism.^138^

### 4.4 Depression

Depression is a major mood disorder, caused mainly by psychiatric disorders, immune dysregulation, genetic factors and environmental, characterized by persistent low mood and recurrent thoughts of death. While it is not entirely clear whether there is a causal relationship between gut microbiota and depression, it has long been recognized that gut microbiota plays a crucial role in the pathogenesis of depression.^78^ An increasing number of studies have shown that the gut microbiomes of healthy individuals and depressed patients differ significantly, mainly in terms of enrichment of pro‐inflammatory bacteria and depletion of anti‐inflammatory bacteria.^139^, ^140^ Furthermore, FMT of depressed patients induced depression‐like behaviour in rodents, suggesting that dysbiosis may be involved in the pathogenesis of depression.^79^ The current study also found that *Mycobacterium spp* was enriched in depressed patients and that *Mycobacterium spp* also significantly increased susceptibility to depression.^141^ In addition, SCFAs were found to be reduced in depressed patients, but exogenous butyrate supplementation may alleviate depression via the HPA axis.^142^

The preceding association studies found a strong link between aberrations in gut microbiota homeostasis and depression; however, they did not provide evidence on aetiology. The findings reveal that MGB axis disruption is key in the pathophysiology of depression.^143^ Notably, overactivation of the HPA axis is one of the causes of depression, and gut microbiota dysbiosis can result in the production of different mediators, such as pro‐inflammatory cytokines and microbial antigens, which activate the HPA axis across the BBB.^144^ Furthermore, gut microbiota dysbiosis increases permeability of the intestinal barriers and BBB, and peripheral pro‐inflammatory cytokines enter the brain and activate microglia, resulting in neuroinflammation.^145^ Neurotransmitters, including 5‐HT, NE and γ‐GABA, are metabolized by gut microbiota and play a crucial role in the formation and plasticity of brain circuits linked to mood disorders including depression. γ‐GABA is the primary inhibitory neurotransmitter in the brain and malfunction has been linked to various neurological disorders such as depression.^146^ Studies have demonstrated that the gut microbiota may generate or deplete GABA, which impacts circulating GABA levels; for example, serum GABA levels are considerably lower in GF animals.^85^

### 4.5 Alterations in the gastrointestinal system in neurological disorders

In conclusion, the interaction between the gut microbiota and the brain plays a pivotal role in the pathogenesis of neurological disorders such as AD, PD, autism and depression. Conversely, numerous clinical observations have demonstrated that patients with neurological disorders frequently exhibit abnormalities in gastrointestinal function and alterations in gut microbiota composition. For example, approximately 40% of autistic patients present with symptoms of gastrointestinal dysfunction, including abdominal cramps, diarrhoea, reflux and vomiting.^147^ Furthermore, a substantial body of evidence from numerous studies indicates that neurological abnormalities, such as stress, depression and overexertion, can disrupt gastrointestinal homeostasis, impair intestinal barrier function and increase intestinal permeability.^148^

Similarly, gastrointestinal dysfunction is prevalent and precedes motor symptoms in patients with PD.^149^ However, current studies are primarily focused on describing and evaluating gastrointestinal symptoms, with fewer studies investigating the pathogenesis of gastrointestinal dysfunction. It is currently believed that gastrointestinal dysfunction in Parkinson's patients is associated with ENS, dorsal motor nucleus of vagus (DMV), gut microecological dysbiosis and intestinal barrier disruption. Studies have demonstrated that the abnormal accumulation of Lewy bodies in the ENS has been observed in the early stages of PD patients, with the potential to reach the CNS through the VN and induce nigrostriatal lesions.^150^, ^151^ Furthermore, the absence of certain enteric neurons and the abnormal activation of enteric glial cells and pro‐inflammatory phenotype are present in patients with PD and animal models.^152^, ^153^ It is worth noting that intestinal microecological dysregulation in PD patients may trigger gastrointestinal function. The abundance of pro‐inflammatory bacteria *Akkermansia*, *Oscillopsia* and *Bacteroides* was shown to be dramatically elevated in faecal samples from PD patients, resulting in local intestine inflammation.^154^ Additionally, changing microbial metabolites alter gastrointestinal function. Elevated levels of p‐cresol and phenylacetylglutamine in metabolites have been found in PD patients, and both are closely linked with the severity of constipation.^155^ There are no specific guidelines for assessing and treating gastrointestinal dysfunction in PD patients, so finding new therapeutic targets that can easily alleviate PD‐induced gastrointestinal dysfunction is critical to improving the disease's progression and patients' quality of life.

## 5 TARGETING GUT MICROBIOTA FOR THE TREATMENT OF NEUROLOGICAL DISEASES

**FIGURE 3:**
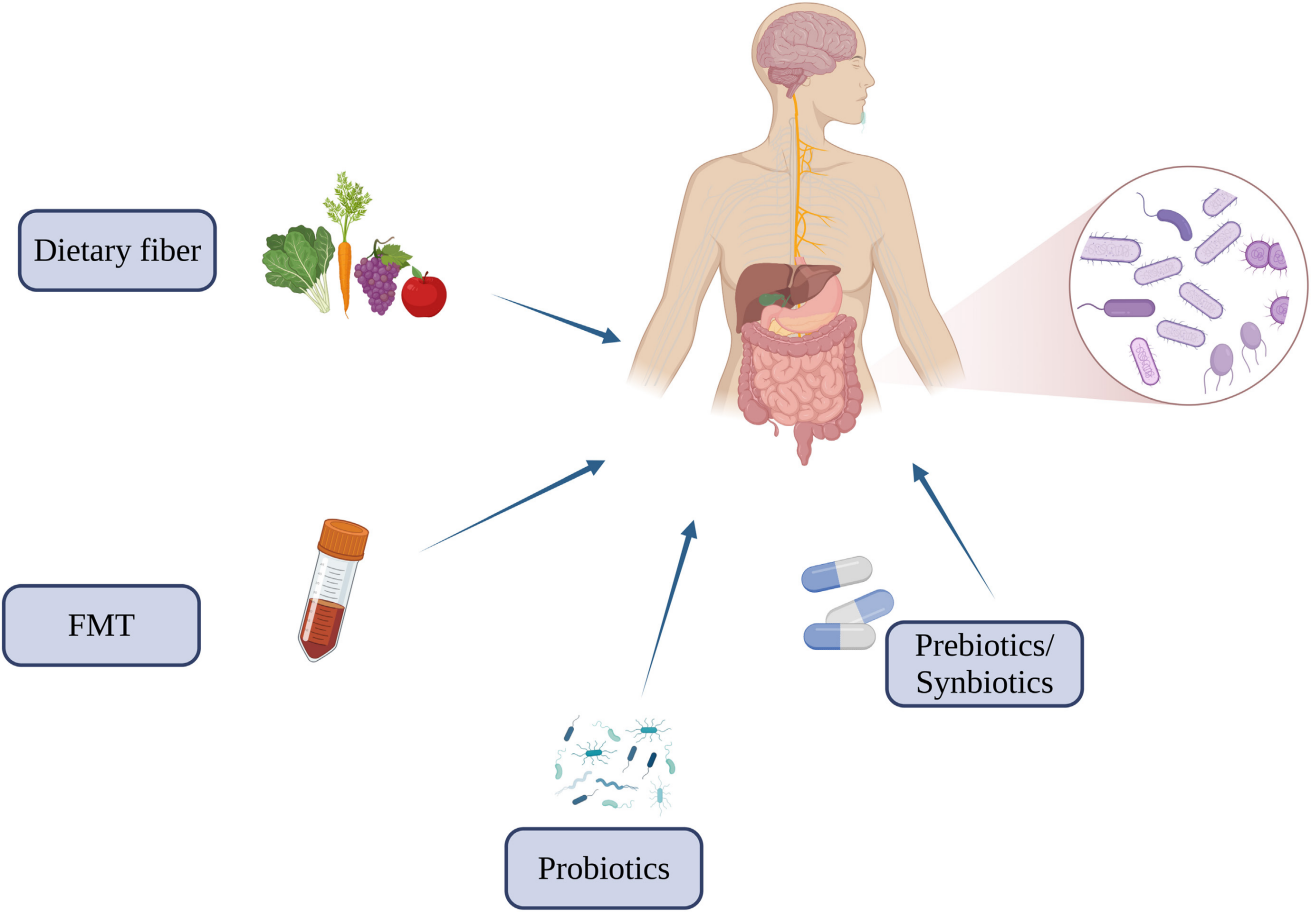
Targeting gut microbiota for the treatment of neurological diseases. Dietary regimens, prebiotics, probiotics and FMT represent potential adjunctive therapeutic strategies for neurological disorders, with the regulation of gut microbiota being a key area of interest. The dietary intake of nutrients and their metabolites has been demonstrated to modulate neuroinflammation, with dietary interventions showing promise in ameliorating the dysfunctions and metabolic disorders commonly seen in neurological disorders. Prebiotics and probiotics can be selectively utilized by host microorganisms and contribute to the creation of a healthy gut environment, which may result in the balancing of the gut microbiota and the production of metabolites, such as organic acids, to maintain organismal health. Furthermore, FMT has been demonstrated to treat central nervous system disorders, including autism and Parkinson's disease. Schematic created with BioRender.com.

Recent findings have shown the potential of microbial interventions in modulating neurological disorders driven by gut ecological dysregulation. Diet, prebiotics, probiotics, synbiotics and FMT are the main strategies for targeting the gut microbiota to treat neurological disorders (Figure 3).

### 5.1 Diet therapy

Diet composition to some extent influences the composition of the gut microbiota, and there is growing evidence that altering the gut microbiota by diet may substantially affect the progression of neurological disorders. For example, a high‐fat diet may increase amyloid angiopathy and oxidative stress, predisposing to cognitive dysfunction and AD^156^, ^157^; in contrast, the Mediterranean diet (MD) may attenuate the progression of AD, and the beneficial effects of the MD may be mediated by the gut microbiota and its anti‐inflammatory properties.^158^, ^159^ In addition, ketogenic diets that are high in fat, adequate in protein and low in carbohydrates may limit glycolysis and increase fatty acid oxidation to produce ketone bodies, which in turn may have a significant effect on ameliorating neurodegenerative diseases.^160^, ^161^

In conclusion, poor dietary intake contributes to gut microbiota dysbiosis, which in turn contributes to neurodegeneration, cognitive decline and anxiety‐like behaviours, while rational dietary interventions can modulate gut microbiota and ameliorate neurodegenerative disorders to some extent. It is worth noting that, in addition to diet, the general lifestyle of patients with neurological disorders requires particular attention. Restrictive diets may play a crucial role in reducing health risks and cardiovascular disease factors in these patients, but highly restrictive diets may also make patients more susceptible to malnutrition by reducing gut microbiota diversity.^162^

### 5.2 Probiotics

Probiotics have been applied to exert anti‐inflammatory effects in intestinal and extra‐intestinal distal organ diseases due to their role in regulating the gut microbiota, enhancing the intestinal barrier and producing beneficial metabolites.^163^ Commonly used probiotics include *Lactobacillus*, *Bifidobacterium*, *Bacillus* and *Enterococcus*. Bonfili et al. first demonstrated that long‐term supplementation with SLAB51, a probiotic preparation of Lactobacillus and *Bifidobacterium*, reduced neuronal protein hydrolysis and Aβ aggregation and attenuated cognitive decline and brain damage in Tg mice through a mechanism that may involve a reduction in oxidative stress through the SIRT3 pathway.^164^ Furthermore, animal‐based studies on probiotic interventions have indicated that probiotics possess a mitigating effect on AD. For example, *Saccharomyces boulardii* has been shown to alleviate neuroinflammation and synaptic damage by regulating the gut microbiota, inhibiting microglial activation and the TLRs pathway and ameliorating cognitive impairment in AD mice.^165^

Clinical trials have demonstrated that probiotics can effectively ameliorate neurological disorders such as depression and autism in rodents by regulating the composition of gut microbiota. However, the complexity of gut microbiota and the difficulty in research methodology have resulted in a paucity of clinical studies on gut microbiota. Furthermore, the mechanisms involved in their metabolism and interactions with each other remain unclear. Additionally, the specificity of probiotic strains, optimal dosage, duration of intervention and other issues vary greatly in preclinical and clinical trials. Consequently, it is imperative to conduct further research and develop new microorganisms with probiotic functions.

### 5.3 Prebiotics/Synbiotics

Prebiotics are currently defined as ‘a substrate that is selectively used by host microorganisms to confer a health benefit’ and are mainly carbohydrates such as inulin, oligo‐galactose, oligo‐fructose and lacto‐fructose, which stimulate the growth and metabolic activity of probiotics in the gut.^165^ For instance, inulin supplementation in mice promotes *bifidobacterial* and *lactobacilli*.^166^ Furthermore, prebiotic bacterial fermentation generates SCFAs, specifically butyrate, acetate and propionate.^166^ Many clinical studies have assessed the impact of prebiotics on alleviating neurological disorders, including AD and PD, although most have concentrated on the beneficial effects of polyunsaturated fatty acids.^167^, ^168^, ^169^ A case in point is the dietary ingestion of omega‐3 fatty acids that might improve cognitive function in mild AD sufferers.^169^ Correspondingly, inulin and oligo‐fructose have shown similar results.^168^, ^169^, ^170^

### 5.4 FMT

Based on the correlation between gut microbiota and neurological disorders, the use of a healthy combination of microbiota in place of a disrupted microbiome may be a potential therapeutic strategy.^171^ FMT is a method of restoring gut microbiota homeostasis by transferring faeces (whole gut microbiota) from a healthy donor to the recipient's gastrointestinal tract. FMT has proven to be the foremost method of treatment for relapsing or refractory *C. difficile* infection and has also been successfully used in gastrointestinal disorders including IBD, IBS, Crohn's disease and ulcerative colitis.^172^ Recent research reveals that FMT might also have an effect on neurological disorders by regulating dysregulated gut microbiota.^15^ Most relevant studies have concentrated on animal testing, with only a handful of clinical studies on human subjects showcasing FMT potential to effectively treat neurological disorders. Research has revealed that FMT minimizes the formation of amyloid plaques and alleviates cognitive impairment in animal models of AD.^173^ Furthermore, a 2018 case study of two ASD patients exhibited significant reduction in symptoms after undergoing FMT treatment.^174^ Moreover, similar findings were observed in paediatric patients with ASD, wherein an improvement in both gastrointestinal and behavioural symptoms as well as microbiome diversity were observed.^175^

FMT may become a new treatment for CNS illnesses; however, the degree of evidence from existing clinical research is severely lacking, and the selection of transplant donors, standardization of transplant samples, efficacy duration and commencement of action must be examined. Furthermore, the characteristics of gut microbiota abnormalities varies between neurological illnesses, and the presence of disease specificity and personalized transplantation methods requires additional investigation.

## 6 CONCLUSION

The precise mechanism by which gut microbiota affect neurological illnesses is still unknown because of the complicated etiopathology of neurological disorders, but a substantial body of research has shown a connection between gut microbiota and brain growth and function. Gut microbiome influences brain development and function by changing its composition and variety, as well as the metabolites it produces, via a bidirectional connection between the gut–brain axis. Recent research points to the possibility of treating neurodegenerative illnesses by focusing on flora, but clinical trials are still required to confirm this. Thus, the pursuit of neuromodulator processes that target MGB and interferable gut targets will remain a popular area of study.

## AUTHOR CONTRIBUTIONS

**Yuhong He:** Conceptualization (equal); visualization (equal); writing – original draft (equal). **Ke Wang:** Data curation (equal); formal analysis (equal); funding acquisition (equal). **Niri Su:** Investigation (equal); supervision (equal); validation (equal); visualization (equal). **Chongshan Yuan:** Investigation (equal); validation (equal); visualization (equal). **Naisheng Zhang:** Funding acquisition (equal); software (equal); writing – review and editing (equal). **Xiaoyu Hu:** Software (equal); supervision (equal); validation (equal). **Yunhe Fu:** Funding acquisition (equal); writing – review and editing (equal). **Feng Zhao:** Conceptualization (equal); formal analysis (equal); supervision (equal); validation (equal).

## FUNDING INFORMATION

The study is supported by the National Natural Science Foundation of China (32122087, 32102738 and 31972749).

## CONFLICT OF INTEREST STATEMENT

No potential conflict of interest was reported by the authors.

## Data Availability

Data sharing not applicable ‐ no new data generated, or the article describes entirely theoretical research.
